# Universal MRSA Decolonization of Critically Ill Patients: An Intervention to Decrease Central Line Associated Bloodstream Infections

**DOI:** 10.1017/ash.2025.293

**Published:** 2025-09-24

**Authors:** Blake Piepenbrink, Jessica Abrantes-Figueiredo, Brenton Nash, Dora Wiskirchen

**Affiliations:** 1 University of Connecticut Primary Care Internal Medicine Residency; 2 Jessica Abrantes-Figueiredo, Saint Francis Hospital, THofNE; 3 Saint Francis Hospital and Medical Center

## Abstract

**Background:** Healthcare associated infections (HAIs) are important areas of concern as they increase length of hospital stay, increase hospital costs, and have high morbidity and mortality. For instance, central line associated bloodstream infections (CLABSIs) approximately increase length of stay by 13.4 days and increase hospital costs by $43,975. Studies also suggest a 1.5-2.5x increase in mortality in patients who develop CLABSIs. In 2013, the REDUCE MRSA trial compared universal MRSA decolonization to targeted MRSA decolonization in the ICU and found superiority ini reducing positive MRSA cultures and all cause bloodstream infections. We aim to decrease central line associated bloodstream infections at our institution by adopting the REDUCE MRSA trial protocol. **Methods and Outcomes:** All patients admitted to the medical/surgical ICU and cardiac ICU at St Francis Hospital starting in December of 2023 received daily intranasal mupirocin and chlorhexidine bathing regardless of their MRSA status. The primary outcome assessed was the CLABSI rate per month. The secondary outcomes were the standard infection ratio and CLABSI per central line day. We compared data from 2020-2023 to data after initiation of the protocol in 2024. We used unpaired t testing to assess the CLABSI rate per month and used a negative binomial regression model to calculate the standard infection ratio according to the NHSN 2015 national baseline. **Results and Discussion:** We had a total of 6 CLABSIs in the ICU this year after initiating universal MRSA decolonization. The number of CLABSIs per month decreased from 0.65 per month from 2020-2023 down to 0.50 per month in 2024. These results, while not statistically significant, are limited by the small sample size since the protocol was just initiated this year. One interesting finding was 5 of the 6 CLABSIs occurred during January through March, which brings up the question if introducing these new changes required time for nursing education and compliance to improve. **Conclusions:** Our results suggest that universal MRSA decolonization in the ICU may decrease the number of CLABSIs. We will continue to collect more data in the coming years to assess for statistical significance. We recommend further research to assess for potential benefits of universal MRSA decolonization in other areas of the hospital where MRSA infection rates are high like step down units.

